# Impact of Antiepileptic Drugs on Cognition and Neuromagnetic Activity in Childhood Epilepsy With Centrotemporal Spikes: A Magnetoencephalography Study

**DOI:** 10.3389/fnhum.2021.720596

**Published:** 2021-09-10

**Authors:** Kai Niu, Yihan Li, Tingting Zhang, Jintao Sun, Yulei Sun, Mingzhu Shu, Pengfei Wang, Ke Zhang, Qiqi Chen, Xiaoshan Wang

**Affiliations:** ^1^Department of Neurology, The Affiliated Brain Hospital of Nanjing Medical University, Nanjing, China; ^2^Department of Neurology, Affiliated Hospital of Jiangsu University, Zhenjiang, China; ^3^MEG Center, The Affiliated Brain Hospital of Nanjing Medical University, Nanjing, China

**Keywords:** magnetoencephalography, cognition, source location, functional connectivity, antiepileptic drug, childhood epilepsy with centrotemporal spikes

## Abstract

**Objective:** Childhood epilepsy with centrotemporal spikes (CECTS), the most common childhood epilepsy, still lacks longitudinal imaging studies involving antiepileptic drugs (AEDs). In order to examine the effect of AEDs on cognition and brain activity. We investigated the neuromagnetic activities and cognitive profile in children with CECTS before and after 1 year of treatment.

**Methods:** Fifteen children with CECTS aged 6–12 years underwent high-sampling magnetoencephalography (MEG) recordings before treatment and at 1 year after treatment, and 12 completed the cognitive assessment (The Wechsler Intelligence Scale for Children). Next, magnetic source location and functional connectivity (FC) were investigated in order to characterize interictal neuromagnetic activity in the seven frequency sub-bands, including: delta (1–4 Hz), theta (4–8 Hz), alpha (8–12 Hz), beta (12–30 Hz), gamma (30–80 Hz), ripple (80–250 Hz), and fast ripple (250–500 Hz).

**Results:** After 1 year of treatment, children with CECTS had increased scores on full-scale intelligence quotient, verbal comprehension index (VCI) and perceptual reasoning index (PRI). Alterations of neural activity occurred in specific frequency bands. Source location, in the 30–80 Hz frequency band, was significantly increased in the posterior cingulate cortex (PCC) after treatment. Moreover, FC analysis demonstrated that after treatment, the connectivity between the PCC and the medial frontal cortex (MFC) was enhanced in the 8–12 Hz frequency band. Additionally, the whole-brain network distribution was more dispersed in the 80–250 Hz frequency band.

**Conclusion:** Intrinsic neural activity has frequency-dependent characteristic. AEDs have impact on regional activity and FC of the default mode network (DMN). Normalization of aberrant DMN in children with CECTS after treatment is likely the reason for improvement of cognitive function.

## Introduction

Childhood epilepsy with centrotemporal spikes (CECTS) is the most common idiopathic focal epilepsy syndrome, and accounts for 13–23% of childhood epilepsy (Heijbel et al., 1975; Panayiotopoulos et al., 2008). In the past, CECTS was called “benign epilepsy with centrotemporal spikes,” however, recent studies have revealed that children with CECTS tend to exhibit extensive cognitive difficulties, which makes the definition of “benign” no longer applicable (Wickens et al., 2017). Therefore, rational treatments of antiepileptic drugs (AEDs) are recommended to control seizures, as well as alleviate cognitive impairment (Hughes, 2010; Mellish et al., 2015). However, the exact long-term effect of AEDs on the intrinsic brain activity and cognitive function among children with CECTS remains unclear.

To date, numerous studies have demonstrated that the resting-state brain activity of children with CECTS has been altered (McGinnity et al., 2017; Bourel-Ponchel et al., 2019; Zhang et al., 2020). On this basis, several studies based on functional magnetic resonance (fMRI) have further investigated brain alterations in CECTS during AEDs treatment, the results of which have shown that AEDs have an effect on functional connectivity (FC) (Jiang et al., 2019, 2020), and regional activity (Zhang et al., 2017, 2018). Unfortunately, these studies are all cross-sectional studies, rather than longitudinal follow-up studies, and lead to limited convincing results. Besides, although the effect of AEDs on cognitive function has been widely reported among previous studies (Tacke et al., 2016; Han and Kim, 2018; Operto et al., 2019), the lack of synchronized cognitive assessment weakens the clinical significance of neuroimaging results.

In addition, increasing evidence suggests that high-frequency oscillations may be a specific biomarker for measuring disease activity, as well as assessing treatment response (van Klink et al., 2016; Frauscher et al., 2017; Lanzone et al., 2020, 2021a). However, fMRI studies only retain low-frequency amplitudes, and may lose high-frequency information of neural activity (Wang et al., 2014). Also, studies that are based on fMRI and electroencephalogram show that brain activity exhibit frequency-dependent properties (Gohel and Biswal, 2015; Jiang et al., 2019; Jun et al., 2019; Lanzone et al., 2021b). Magnetoencephalography (MEG) is a non-invasive technique with high spatiotemporal resolution that records brain activity by detecting magnetic fields generated by neural currents (Guggisberg et al., 2008; Burgess, 2011). These magnetic fields are not attenuated by the scalp and skull, allowing MEG to detect the higher frequency electromagnetic oscillations of the brain (Guggisberg et al., 2008; Burgess, 2011; Harmsen et al., 2018). Furthermore, compared to other neuroimaging techniques, MEG has several advantages for use among young children, including fewer constraints and the absence of radiation and unpleasant sounds (Kikuchi et al., 2016). Overall, MEG is known to be suitable for multifrequency analysis of brain activity among children with CECTS.

In this study, we utilized MEG to directly compare the neuromagnetic signatures and FC network before and after drug treatment from low- to high-frequency bands in order to explore the changes in brain activity of children with CECTS that receive AEDs. We also simultaneously performed cognitive assessment to link cognitive profile changes that are caused by AEDs with neuroimaging results. Our finding likely contributed to gain better insight into the neural mechanisms of AEDs on CECTS therapy.

## Experimental Procedures

### Participants

Children that are diagnosed with CECTS were recruited from the Department of Neurology at Nanjing Children’s Hospital and Nanjing Brain Hospital between November 2018 and November 2019. All children started taking AEDs within 1 week after completing the initial MEG recording, and performed the second recording after a period of treatment. The time interval was 8.8–18.3 months (average 11.9 months).

The inclusion criteria for subjects were as follows: (1) a clinical diagnosis of CECTS, according to the ILAE 2017 classification of epilepsy syndrome (Scheffer et al., 2017); (2) normal brain MRI, no history of other neurological diseases or major diseases; (3) 6–12 years old, undergoing normal development and receiving formal education; (4) drug naive at enrollment; and (5) requirement of drug intervention as judged by an experienced neurologist.

The exclusion criteria are as follows: (1) a history of taking AEDs, (2) metal implants within the body, (3) atypical evolution, (4) interruption of treatment or combined use of multiple drugs during follow-up, and (5) lost to follow-up.

### MEG Recording

A whole-head, CTF-275 channel MEG system (VSM Medical Technology Company, Canada) was utilized to collect the MEG data in a magnetically shielded room. The MEG date was collected at a sampling rate of 6000 Hz, with noise cancelation of the third-order gradients. Prior to MEG recording, three small electromagnetic coils were pasted on the nasion and bilateral pre-auricular points of each subject, which helped monitor the subject’s head position relative to the MEG sensors. The accuracy of the head position was 1 mm. During the recording, subjects were instructed to relax, stay still, gently close their eyes and slightly open their mouths. Each MEG recording lasted for 2 min. Data with head movement >5 mm was discarded so as not to affect accuracy of the source localization. For each subject, at least ten valid data files for a total of 20 min were collected.

### MRI Scan

The MRI date was obtained on a 3.0T MRI scanner (Siemens, Germany). The T1-weighted images were acquired using the following parameters: sagittal orientation, slices = 176, thickness = 1 mm, TR = 1,900 ms, TE = 2.48 ms, matrix = 512 × 512, and field of view = 250 mm × 250 mm. Prior to scanning, three fiduciary marks were also pasted on positions of the coils in the MEG recording, in order to accurately co-register the MRI and MEG data.

### Data Preprocessing

We performed data processing via the following steps: First, we deleted the data with discernible noise and artifacts (waveform > 6pT deflection) by visually inspecting the MEG waveform (Xiang et al., 2015b). Then, we filtered the remaining MEG data with a band pass filter of 1–70 Hz so that the characteristic spikes can be clearly displayed in the waveform. Next, we selected a segment of interictal waveform with no spikes for up to 60 s. Finally, we analyzed the selected waveform segments in the following seven frequency bands, including: delta (1–4 Hz), theta (4–8 Hz), alpha (8–12 Hz), beta (12–30 Hz), gamma (30–80 Hz), ripple (80–250 Hz), and fast ripple (250–500 Hz). Notch filters for 50 Hz and its harmonics were utilized to eliminate the interference from ambient alternating current power.

### Source Localization

Based on the previous studies (Xiang et al., 2014, 2015b), we utilized accumulated source imaging (ASI), which is a specific approach to analyze neuromagnetic activity among multiple frequency ranges, as well as to analyze MEG signals at the source level. In brief, ASI is the volumetric summation of source activity over a period of time (Xiang et al., 2015b), which can be depicted by the following equation:


(1) $$\mathrm{Asi}\left(r,s\right)=\sum_{t=1}^{t=n}Q\left(r,t\right)$$

Where Asi is accumulated source strength at location r, s is the time slice, t is the time point of MEG data, n is the total time points of MEG data and Q is the source activity at source r and at time point t. We defined s ≥ 1 and s ≤ n/2.

We used two-step beamforming to localize the magnetic sources (Xiang et al., 2015b). Specific steps were as follows. First, we computed the lead fields for each source (or voxel position) to generate matrices with MEG data. The next crucial step was selecting sensors for partial sensor coverage for each voxel, which was done to minimize the effect of coherent sources in source localization (Xiang et al., 2015a). Then, we computed the covariance for all sensors. Furthermore, we computed two sets of magnetic source images using a 3D vector beamformer grid, and estimated the coherent source and source orientation with a covariance matrix vector beamformer (Xiang et al., 2015a). Finally, we generated source activity (or virtual sensor waveform) using a scalar beamformer (Xiang et al., 2015a). The detailed mathematical algorithms and validations were described in the previously published articles (Xiang et al., 2014, 2015b).

### Functional Connectivity Analysis

We analyzed FC at the source level, and specific procedures and algorithms of this method were elaborated in the previous reports (Xiang et al., 2014, 2015b). In summary, the virtual sensor waveforms of each source were calculated using the ASI algorithm mentioned above. Next, the source neural network was estimated via analysis of the signal correlation of each pair of virtual sensors in the 60 s time window. Finally, the relationship between the virtual sensor signals from the two source pairs was statistically analyzed by calculating the correlation factor (or the correlation coefficient). The correlation factor is calculated via the following formula:


(2) $$R\left(\mathrm{Xa},\mathrm{Xb}\right)=\frac{C\left(\mathrm{Xa},\mathrm{Xb}\right)}{\mathrm{SXa},\mathrm{Xb}}$$

Where R (X_a_, X_b_) represent the correlation between the pair of magnetic sources “a” and “b,” while X_a_ and X_b_ represent signals from two of the magnetic sources calculated in pairs. C (X_a_, X_b_), respectively, represent the average signals of the two magnetic sources, while SX_a_ and SX_b_ are the standard deviations of signals of the two magnetic sources.

In order to avoid possible bias, source-level analysis was utilized to calculate all possible connections for each source pair. All possible FC distributions of each pair of voxel-based virtual sensors were co-registered to MRI of each participant (Xiang et al., 2014, 2015b). In order to analyze source connections, the neural network was visualized in axial, coronal, and sagittal views. Excitatory and inhibitory connections were depicted in red and blue, respectively. In order to ensure the quality of the data, a threshold was utilized as a checkpoint. *T* values were computed for all source pairs to determine the thresholding of connections.


(3) $$\mathrm{Tp}=R\sqrt{\frac{K-2}{1-R^{2}}}$$

Where T_p_ is the *t*-value of a correlation, R is the correlation of a source pair, and K is the number of data points for the connection. In this study, we used a threshold of a T_p_ value with a corresponding *p-*value < 0.05 to obtain the FC networks.

### Neurocognitive Assessment

The Wechsler Intelligence Scale for Children, fourth edition (WISC-IV) is a standardized test designed to measure intelligence of children and adolescents from 6 to 16 years old. The scale consists of 10 subscales core subtests and five additional subtests through which it is possible to calculate one full-scale intelligence quotient (FSIQ) and four indices. The details are as follows: The verbal comprehension index (VCI) represents language and verbal skills, the perceptual reasoning index (PRI) reflects nonverbal and fluid reasoning, the working memory index (WMI) reflects working memory and auditory attention, and the processing speed index (PSI) reflects selective attention and speed of visual information processing. We previously reported that, prior to treatment, children with CECTS showed significantly poorer performance compared to health controls in all aspects (Li et al., 2020a). The WISC-IV was utilized in the present study to determine whether treatment with AEDs would cause a change in cognitive function.

### Statistical Analyses

The McNemar’s test was conducted on predominant neuromagnetic source locations and FC network patterns. For WISC-IV scores, statistical analyses were carried out using a paired sample *T*-test after demonstrating normal distribution with a Shapiro–Wilk test. Bonferroni correction was utilized for multiple comparisons [i.e., for seven frequency bands, *p* < 0.007 (0.05/7 = 0.00714)]. Probability values < 0.05 (two-tailed) were considered to be statistically significant. All statistical analyses were conducted using SPSS version 23.0 for Windows (SPSS Inc., Chicago, IL, United States).

## Results

### Clinical Characteristics

A total of 22 children were recruited for this study. Among them, children who were lost to follow-up (*n* = 5) and children with atypical evolution (*n* = 2) were excluded from the study. Data from the remaining 15 patients (eight female and seven male) were analyzed. Their average age of onset was 7.65 ± 1.44 years. Their average course of disease was 6.40 ± 3.92 months at the time of enrollment, and 18.33 ± 5.45 months at the time of follow-up. The average follow-up interval is 11.93 ± 2.80 months. Seven children no longer experienced seizures after treatment, while the other eight continued to have seizures. Detailed data is described in Table 1.

**TABLE 1:** Demographic and clinical characteristics of the patients.

Patients	Onset age, y	Sex	Seizure type	Seizure frequency	Duration, m		Initial AED	Maintenance Dose, mg	Seizure controlled
					Enrollment	Follow-up			
1	6.95	F	FS with PA	Four episodes	8.9	22.6	LEV	1000	Y
2	4.64	M	Focal to bilateral TCS	Three episodes	9.7	22.9	VPA	750	N
3	7.93	F	Focal to bilateral TCS	Weekly	4.6	15.8	LEV	1750	N
4	4.43	M	FS with IA	Four episodes	10.9	20.3	LEV	750	Y
5	9.65	F	Focal to bilateral TCS	One episode	5.6	14.7	LEV	750	N
6	9.46	M	Focal to bilateral TCS	Two episodes	6.1	24.4	LEV	1000	N
7	8.89	M	Focal to bilateral TCS	Three episodes	0.5	9.3	OXC	450	Y
8	8.25	F	FS with PA	Two episodes	5.7	16.5	OXC	600	N
9	7.23	F	FS with IA	Two episodes	9.5	25.1	LEV	1250	N
10	9.60	F	FS with IA	Three episodes	7.7	16.6	OXC	750	N
11	7.18	M	Focal to bilateral TCS	Two episodes	1.3	11.9	VPA	500	Y
12	8.93	M	FS with PA	Four episodes	14.5	28.7	LEV	1500	Y
13	5.15	F	FS with IA	Four episodes	4.5	15.5	OXC	450	Y
14	7.30	F	FS with PA	Two episodes	0.8	13.4	LEV	1000	Y
15	6.21	M	Focal to bilateral TCS	Three episodes	5.7	17.3	OXC	600	N

*F, female; M, male; FS, focal seizures; PA, preserved awareness; IA, impaired awareness; TCS, tonic-clonic seizures; AED, antiepileptic drug; LEV, levetiracetam; VPA, valproic acid; OXC, oxcarbazepine; Y, yes; N, no.*

### The WISC-IV Score

**FIGURE 1:**
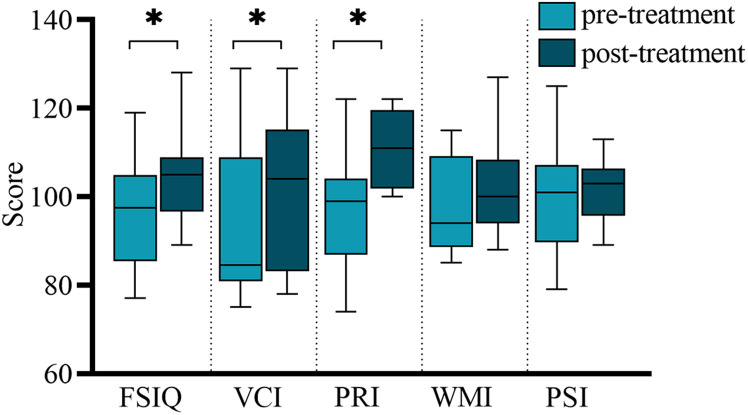
Comparison of WISC-IV scores before and after treatment. FSIQ, Full-scale Intelligence Quotient; VCI, verbal comprehension index; PRI, perceptual reasoning index; WMI, working memory index; PSI, processing speed index. ^∗^The *p*-value was statistically significant.

In total, 12 children completed the two tests at the time of enrollment and follow-up. Comparing the mean scores before and after treatment, we discovered found that treatment led to better scores in FSIQ, VCI, PRI, WMI, and PSI. The improvement in FSIQ, VCI and PRI was found to be statistically significant. Table 2 and Figure 1 summarizes the mean scores of the two times.

**TABLE 2:** WISC-IV scores before and after treatment.

WISC-IV	Pre-treatment	Post-treatment	Difference	*P*-value
FSIQ	96.08 ± 11.91	105.08 ± 10.00	9.00 ± 11.40	0.019*
VCI	93.42 ± 17.19	101.33 ± 16.54	7.92 ± 9.46	0.014*
PRI	97.33 ± 15.31	110.33 ± 8.65	13.00 ± 17.71	0.027*
WMI	98.00 ± 11.32	102.00 ± 11.10	4.00 ± 14.21	0.350
PSI	100.42 ± 12.68	101.75 ± 6.80	1.33 ± 13.51	0.739

FSIQ, Full-scale Intelligence Quotient; VCI, verbal comprehension index; PRI, perceptual reasoning index; WMI, working memory index; PSI, processing speed index. *The p-value was statistically significant.

### Source Localization

**FIGURE 2:**
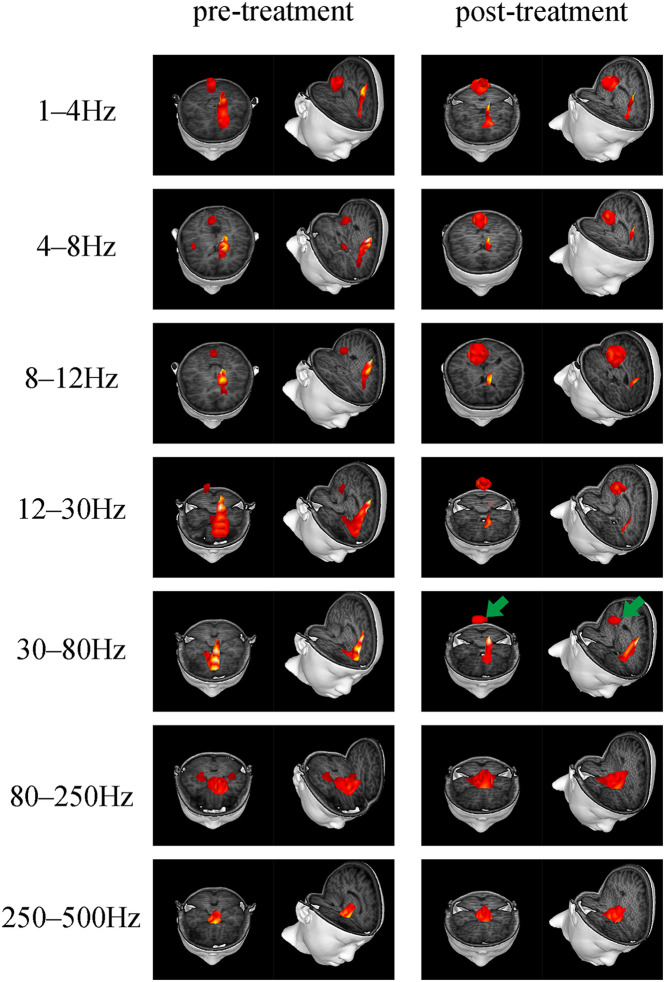
Typical predominant magnetic source locations in the 1–500 Hz frequency range before and after treatment. Activation of the PCC in the 30–80 Hz frequency band increased after treatment, which is indicated by green arrows.

Accumulative magnetic source imaging demonstrated that each patient has 1–3 sources stronger than the rest of the brain activity during the resting state. Regarding the location of the main magnetic source (Figure 2), we discovered similar patterns were present before and after treatment. Specifically, in the 1–4, 4–8, and 8–12 Hz frequency bands, the main magnetic source was found to be located in the medial frontal cortex (MFC), posterior cingulate cortex (PCC), and peri-Rolandic area (PR). In the 12–30 Hz frequency band, the main magnetic source was located in the MFC and PCC. We did not find any significant difference in the magnetic source location before and after treatment in these frequency bands. In the 30–80 Hz frequency band, the main magnetic source was located in the MFC, PCC, and medial temporal lobe (MTL). The brain activity before and after treatment was significantly different at this frequency band, while activation of the PCC after treatment was higher compared to before treatment (*P* = 0.004). In the 80–250 and 250–500 Hz Frequency bands, the main magnetic sources were located in the MFC, MTL, and deep brain area (DBA). Changes in these frequency band were found not to be statistically significant. Detailed statistical results were shown in Table 3.

**TABLE 3:** Predominant source locations before and after treatment.

Frequency band (Hz)	Source location	Pre-treatment	Post-treatment	*p*-value
1–4 Hz	PR	6	4	0.625
	PCC	6	8	0.625
	MFC	13	14	1.000
	MTL	1	0	1.000
	DBA	0	0	–
4–8 Hz	PR	6	4	0.625
	PCC	7	8	1.000
	MFC	14	15	1.000
	MTL	0	0	–
	DBA	0	0	–
8–12 Hz	PR	1	2	1.000
	PCC	7	11	0.219
	MFC	13	9	0.219
	MTL	0	0	–
	DBA	0	0	–
12–30 Hz	PR	0	1	1.000
	PCC	5	10	0.065
	MFC	14	14	1.000
	MTL	1	0	1.000
	DBA	2	1	1.000
30–80 Hz	PR	1	0	1.000
	PCC	2	11	0.004**
	MFC	13	14	1.000
	MTL	4	4	1.000
	DBA	2	1	1.000
80–250 Hz	PR	0	0	–
	PCC	0	0	–
	MFC	10	7	0.508
	MTL	10	11	1.000
	DBA	6	9	0.289
250–500 Hz	PR	0	0	–
	PCC	0	0	–
	MFC	10	9	1.000
	MTL	11	11	1.000
	DBA	7	10	0.250

PR, peri-Rolandic area; PCC, posterior cingulate cortex; MFC, medial frontal cortex; MTL, medial temporal lobe; DBA, deep brain area. **The p-value was statistically significant after Bonferroni correction.

### Functional Network

**FIGURE 3:**
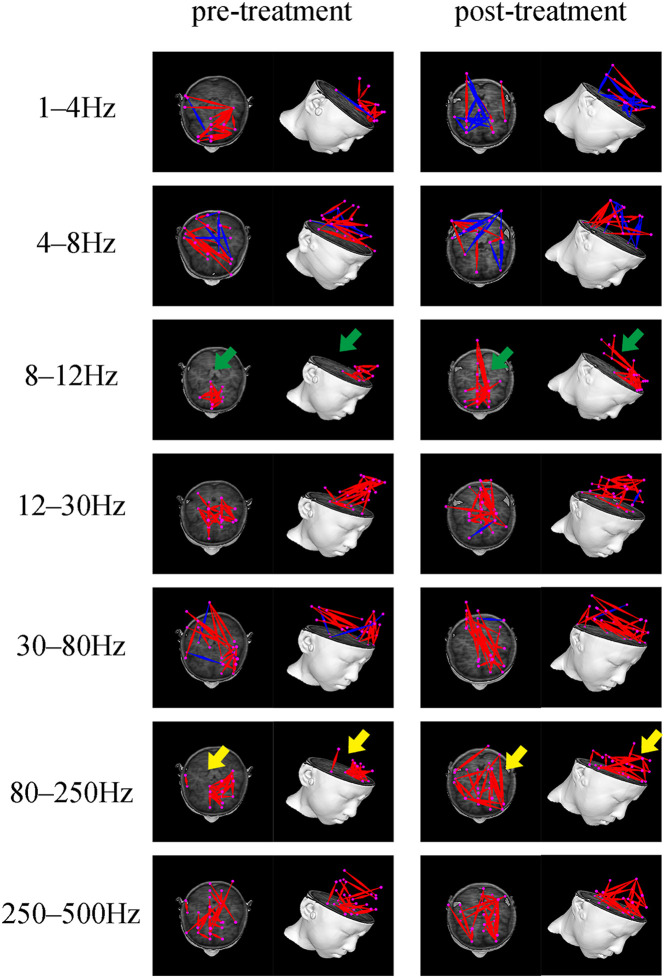
Typical FC networks patterns in the 1–500 Hz frequency range before and after treatment. The FC network patterns before and after treatment are significantly different in the 8–12 and 80–250 Hz frequency bands. The green arrow indicates increased anterior-posterior connection (mainly the connection between the MFC and PCC) after treatment. The yellow arrow indicates a more dispersed whole-brain network distribution pattern after treatment.

Both positive and negative connections of seven frequency bands before and after treatment was analyzed and compared. We found that there were differences in the frequency bands of 1–4, 8–12, and 80–250 Hz. Among them, in the 1–4 Hz frequency band, the functional connection involving the PR tended to decrease post-treatment, compared to before treatment. The *P*-value (*P* = 0.031), however, was not statistically significant after Bonferroni correction. In the 8–12 Hz frequency band, the functional connection between the anterior and posterior brain regions (mainly the connection between the MFC and the PCC) was significantly enhanced after treatment (*p* = 0.001, *p* < 0.05 after Bonferroni correction). In the 80–250 Hz frequency band, the functional network was limited to the MFC before treatment, while it involved more brain regions after treatment (*p* = 0.004, *p* < 0.05 after Bonferroni correction). No statistically significant difference emerged in other frequency bands. Figure 3 demonstrates the typical functional network under untreated and treated conditions.

## Discussion

Herein, we investigated intrinsic neural activity at the source level from both local and global aspects in CECTS patients prior to and after AEDs treatment, providing novel insights into mechanisms of drug treatment. The results demonstrate that neuromagnetic activity exhibited great differences between the various frequency bands, but relative stability before and after treatment on the same frequency band. Notably, in specific frequency bands, children administered AEDs for 1 year seemed to present with increased neuromagnetic activity in the PCC, increased FC involving the default mode network (DMN), as well as altered distribution of the whole brain network. Moreover, we showed that the cognitive profile of children with CECTS is affected by AEDs, as children have better performance on verbal comprehension and perceptual reasoning post-treatment. Thus, changes in regional neuromagnetic activity and whole-brain functional networks based on analysis of multiple frequency bands have potential significance as an imaging biomarker for clinical drug treatment and cognitive development.

We illustrated how cognitive functions in children with CECTS evolve over 1 year of antiepileptic treatment. After treatment, the children performed significantly better on verbal comprehension and perceptual reasoning, and had stable scores on working memory and processing speed. Thus, our results indicate that AEDs do not cause deterioration of cognitive function among children with BECT, which shows good consistency with previous studies (Tacke et al., 2016; Operto et al., 2019). In addition, according to a report (Garcia-Ramos et al., 2015), without drug intervention, children with CECTS show only mild cognitive improvement over time, and still retain deficits. Therefore, we cautiously infer that the improved cognitive response after 1 year of medication in this study is likely attributable to treatment, and not to the natural course of the disease. This positive cognitive trajectory induced by AEDs has also been observed in other different childhood epilepsies. In mixed samples, the neutral-to-beneficial effects of lamotrigine on cognition and vigilance of children with epilepsy during long term treatment has been validated (Brodbeck et al., 2006). Furthermore, a study on childhood absence epilepsy also suggests that AEDs likely improve children’s neurocognitive functions in attention, fine-motor fluency, and visual memory by reducing seizures (Sirén et al., 2007). Although there are differences with regards to research results based on diverse AEDs (Moavero et al., 2017; Han and Kim, 2018), collectively, these findings question the long-standing view that chronic cognitive deficits in children with epilepsy are largely caused by drugs.

Our neuromagnetic findings reveal the frequency-dependent characteristic of intrinsic neural activity in CECTS, which indicates that the source locations vary with frequency bands, exhibiting obvious differences and regularities, both before and after treatment. In brief, the higher the frequency band, the more concentrated the source locations in the deep brain regions. This further supports the theory that neuromagnetic signals of different frequency bands represent distinct inherent physiological activities (Tenney et al., 2014; Tang et al., 2016; Zhang et al., 2020).

In agreement with fMRI evidence based on amplitude of low frequency fluctuation (Jiang et al., 2020) and functional covariance connectivity (Jiang et al., 2019), our results suggest that AEDs have effects on brain regional activity and functional networks. The analysis of the magnetic source location exhibited that, after treatment, activation of PCC increased in multiple frequency bands, and the differences were evident in the 30–80 Hz frequency band. Interestingly, our exploration of the global brain network also demonstrated changes involving DMN, with increased FC between the MFC and PCC after treatment in the 8–12 Hz frequency band. It is well known that MFC and PCC are core regions in the DMN (Buckner et al., 2008; Buckner and DiNicola, 2019), which suggests that DMN may be a potential key target for drug therapy.

The brain is a large-scale network which dynamically regulates information interaction between various systems. The DMN is considered to be a fundamental network for maintaining baseline state of the nervous system, which is inhibited during the working state or in response to significant external stimuli, and resumes and remains active during the resting state (Lin et al., 2016). The proper operation of DMN is very important to the maintenance of normal neurophysiological functions, and many neuropsychiatric diseases are thought to be related to abnormal activation pattern of DMN (Greicius et al., 2004; Ibarretxe-Bilbao et al., 2011; Washington et al., 2014). There is now accumulating evidence that abnormal DMN exists among children with BECT (Oser et al., 2014; Ofer et al., 2018) and plays a pivotal role in the occurrence and development of cognitive and behavioral disorders (Gusnard et al., 2001; Oser et al., 2014). Functionally, DMN can be separated into anterior DNM, which is centered around the MFC, and posterior DMN, which is centered on the PCC (Buckner et al., 2008; Raichle, 2015). The execution of different physiological functions depends on the interaction between the sub-networks (Buckner et al., 2008; Buckner and DiNicola, 2019). Herein, children with BECT demonstrated reduced FC between anterior DMN and posterior DMN prior to treatment. It is intriguing that reduced connectivity between PCC and bilateral MFC is consistently demonstrated in CAE, another childhood epilepsy (Luo et al., 2011). Moreover, reduced connectivity between the anterior and posterior areas of the DMN is related to the duration of epilepsy (McGill et al., 2012). The enhanced activation of PCC and increased FC within DMN after treatment are, therefore, likely to reflect repair or compensation of damaged DMN. This is completely consistent with the view of a previous cross-sectional study (Zeng et al., 2015). That is, due to intervention of AEDs, abnormal brain function in the DMN of children with CECTS has been reversed. Additionally, given that PCC and DMN are closely related to cognitive processes such as remembering and making social inferences (Buckner and DiNicola, 2019), we speculated that normalization of DMN after treatment is a basis for cognitive improvement.

Furthermore, our results indicated that in the 80–250 Hz frequency band, compared to the functional network pattern confined to the MFC before treatment, brain networks of the BECT children after 1 year of treatment tend to be more decentralized and involve more brain regions. This may reflect compensation for the integration of high-frequency information. In the future, this specific mechanism needs to be further explored. In addition, although the results of the 1–4 Hz frequency band in this study were not statistically different after multiple corrections, it is worth noting that previous EEG study of temporal lobe epilepsy also showed similar results, that is, slow wave activity reduces after initial AED therapy (Ricci et al., 2021). In focal epilepsy, the enhancement of slow wave activity is considered to be related to the activity of epileptic focus (Pellegrino et al., 2017). Therefore, the reduction in slow frequency can be explained by the effect of AEDs on epileptic activity and cortical excitability, which is a manifestation of the normalization of brain activity (Ricci et al., 2021). We noticed that the alteration of source location and FC occurred in different frequency bands, respectively. This phenomenon can be attributed to the weak statistical power that is caused by a small sample. Another reasonable explanation can be that different types of neural activities and information processing require different frequencies (Cha et al., 1991; Lopes et al., 2003; Palva and Palva, 2018).

Several limitations to our research exist. First, the number of participants who completed the follow-up was less than expected, and the small sample size weakened the statistical power and the generalizability of the conclusions, to some extent. Meanwhile, this study design was not able to compare brain activity between untreated CECTS children and matched healthy controls, so while several previous studies have reported comparative results for this purpose among untreated children and healthy controls (Jiang et al., 2019, 2020; Li et al., 2020b), we still cannot assert that all the cognitive improvements can be attributed to AEDs. Additionally, this study, limited by sample size, did not group the types of AEDs and the prognosis of the children, which can cause mixed effects. Whether different types of AEDs have different effects on neural activity, or whether there are differences in the brain network patterns among children with different outcomes needs to be clarified in future studies.

## Conclusion

This longitudinal study investigated the effects of long-term use of AEDs on brain activity and cognitive function among children with CECTS. We demonstrated that compensation or normalization of DMN caused by AEDs likely contributes to the improvement of cognition. The dispersed distribution of the whole brain network may be a compensation of AEDs for integration of brain function. The above-mentioned alterations occur in specific frequency bands, which supports the frequency-dependent characteristic of neural activity, and proves the feasibility and effectiveness of MEG technology based on multi-frequency analysis in epilepsy research. Overall, our findings provide novel insights into the effects of AEDs on abnormal brain activity and cognitive deficits among CECTS children.

## Data Availability Statement

The original contributions presented in the study are included in the article/supplementary material, further inquiries can be directed to the corresponding author.

## Ethics Statement

The studies involving human participants were reviewed and approved by the Medical Ethics Committee of Nanjing Brain Hospital. Written informed consent to participate in this study was provided by the participants’ legal guardian/next of kin. Written informed consent was obtained from the individual(s), and minor(s)’ legal guardian/next of kin, for the publication of any potentially identifiable images or data included in this article.

## Author Contributions

KN, YL, and TZ designed the study. KN, YL, TZ, PW, and QC acquired the raw data. JS, YS, MS, and KZ analyzed the data. KN wrote the manuscript. XW revised the manuscript. All authors read and approved the final submitted manuscript.

## Conflict of Interest

The authors declare that the research was conducted in the absence of any commercial or financial relationships that could be construed as a potential conflict of interest.

## Publisher’s Note

All claims expressed in this article are solely those of the authors and do not necessarily represent those of their affiliated organizations, or those of the publisher, the editors and the reviewers. Any product that may be evaluated in this article, or claim that may be made by its manufacturer, is not guaranteed or endorsed by the publisher.

## Abbreviations

CECTS: childhood epilepsy with centrotemporal spikes
AEDS: antiepileptic drugs
FC: functional connectivity
DMN: default mode network
fMRI: functional magnetic resonance
MEG: magnetoencephalography
ASI: accumulated source imaging
WISC-IV: Wechsler Intelligence Scale for Children, fourth edition
FSIQ: full-scale intelligence quotient
VCI: verbal comprehension index
PRI: perceptual reasoning index
WMI: working memory index
MFC: medial frontal cortex
PCC: posterior cingulate cortex
PR: peri-Rolandic area
MTL: medial temporal lobe
DBA: deep brain area.
